# Difructose Anhydride and Passive Immunity Effects on Passive Immune Transfer and Performance of Feeding Difructose Anhydride to Neonatal Calves

**DOI:** 10.3390/ani14010035

**Published:** 2023-12-21

**Authors:** Miguel Escartín, Núria Rialp, Alex Bach

**Affiliations:** 1 Blanca from the Pyrenees, Hostalets de Tots, 27795 Lleida, Spain; miguel@blancahub.cat (M.E.); nuria@blancahub.cat (N.R.); 2 ICREA, Passeig de Lluís Companys 23, 08007 Barcelona, Spain

**Keywords:** antibodies, immunity, growth

## Abstract

**Simple Summary:**

Failure passive transfer of immunoglobulins from colostrum to the calf is a relatively common problem with a prevalence around 20–25%. This failure often leads to impaired health and increased mortality. Many studies have proposed different management and feeding methods to minimize this failure. One of these methods includes the supplementation of difructose anyhdride III (DFAIII). Herein, we evaluate the potential of DFAIII to improve the transfer of immunoglobulins, and in turn animal performance and health, of calves that consumed colostrum with a high immunoglobulin concentration. We conclude that even when feeding calves colostrum with a high immunoglobulin concentration, supplementation with DFAIII may promote passive transfer of immunoglobulins in neonatal Holstein calves during the first 12 h of life.

**Abstract:**

The objective of this study was to assess the potential effects of supplementing difructose anyhdride III (DFAIII) during the first days of life on the absorption of immunoglobulin G (IgG) and growth performance of calves early in life fed colostrum with a high IgG concentration. Sixty-six healthy new-born Holstein calves were randomly assigned to three treatments consisting of no supplementation (control), supplementation of 12 g/d (DFA12), or 36 g/d (DFA36) of DFAIII during the first 7 d of life via colostrum and milk replacer (MR). Calves were separated from dams at birth and bottle-fed colostrum in two meals, each targeting 2.5 L within the first 18 h of birth. Colostrum had been previously collected from other dams (and preserved frozen) within the first 2 h of calving and had a Brix value ≥32%. Daily consumption of starter concentrate and MR (and colostrum on the first day) were individually monitored. Calves were body weighed using an electronic scale at birth and on a weekly basis thereafter until the end of study at 42 d of age. A sample of colostrum fed to each calf and a blood sample from the jugular vein of the calves were collected at 12 and 24 h of life to determine the IgG concentration. The mean colostrum IgG concentration fed in the current study was 110 ± 33.7 g/L (mean ± SD). No differences in animal performance were found among the treatments. Calves on all treatments consumed the same amount of colostrum with a similar concentration of IgG, and thus the amount of IgG consumed was also similar. Serum IgG concentrations were greater at 24 than at 12 h but did not differ among treatments. However, the apparent efficiency of absorption of colostral immunoglobulins was greater in DFA12 and DFA36 at 12 h of life than in control calves, with no differences observed at 24 h. Even when feeding high-quality colostrum, in terms of IgG concentration, supplementation with difructose anhydride III may pose an additional advantage in promoting the passive transfer of immunoglobulins in neonatal Holstein calves during the first 12 h of life.

## 1. Introduction

Calves are born depleted of serum antibodies and they depend on the consumption of colostrum to build some initial degree of passive immunity. Failure passive transfer of immunoglobulins is a relatively common problem in dairy production with a prevalence around 20–25% in different parts of the world [1,2,3]. The consequences of an inadequate passive transfer of immunoglobulins are impaired health and increased mortality [4]. Several studies have evaluated the possibility of improving the passive transfer of immunoglobulins in calves through colostrum management [5], time of feeding relative to birth [6], feeding amounts [7,8], feeding frequency [9], and feeding methods [10,11,12]. Some studies have also attempted to use some additives to foster antibody absorption. For example, ref. [13] reported an increased rate of IgG absorption when supplementing colostrum with mannan-oligosaccharides. Kamada et al. [14] found increases in IgG absorption when supplementing colostrum with selenium and Morrill et al. [15] reported improvements in IgG absorption when colostrum was supplemented with sodium bicarbonate. The intestine of a neonatal calf has no selective absorption capacity for large proteins [16], and molecules of different molecular weight are absorbed more or less at the same rate [17,18], with small differences among the different types of immunoglobulins. Transport of IgG through micropinocytic transfer was demonstrated using electromicroscopy [19]. Previous studies have described that a crystal disaccharide obtained from inulin of chicory root formed by two fructoses and called difructose anhydride III (DFAIII) is able to loosen intestinal tight junctions by changing the distribution of actin filaments and claudin-1 [20], resulting in a reduction in transepithelial electrical resistance [21]. Three previous studies [22,23] reported different degrees of improvements in serum IgG concentrations in calves supplemented with DFAIII, and suggested that the absorption of IgG by endocytosis as well as a non-selective concentration gradient process of the paracellular pathway of the intestinal epithelium could be enhanced by DFA III. The three former studies evaluated the effect of DFAIII on the colostral IgG absorption of calves fed two meals of 2 L of colostrum within the first 24 h of life containing relatively low amounts IgG (<70 g/L). The hypothesis of this study was that supplementation of DFAIII during the 7 d of life would further improve IgG absorption even when fed high-quality colostrum (>100 g of IgG/L). The objectives were to determine the effect of DFAIII on passive transfer of immunoglobulins of high-quality colostrum to neonatal calves.

## 2. Materials and Methods

All the experimental procedures used in this study were evaluated by the ethical committee of the Institut de Recerca i Tecnologia Agroalimentàries (Barcelona, Spain) and approved by the Catalan government under the code 11117b. Sixty-six healthy new-born calves were selected and randomly allocated to 3 treatments (*n* = 22) at birth following a complete block randomized design. All calves were born in the same farm where the experiment was conducted (Blanca from the Pyrenees, Hostalets de Tost, Spain). There was a total of 6 blocks, which included births across a fortnight (11 calves per block). Treatments consisted of either no DFAIII (control), supplementation of a total of 12 g of DAFIII/calf split in 6 g of DFAIII per colostrum or milk replacer (MR) feeding until day 7 of life (DFA12), or 36 g of DAFIII/calf split in 18 g of DFAIII per colostrum or MR feeding until day 7 of life (DFA36). Difructose anhydride III was directly added (6 or 18 g) and mixed with colostrum or MR in individual bottles before every feed. Calves were separated from dams at birth and bottle-fed high-quality colostrum in 2 meals, each targeting 2.5 L within the first 18 h of birth. Colostrum had been previously collected from other dams from the experimental farm within the first 2 h of calving and only those that had a Brix value ≥ 32% were retained and frozen. This colostrum was thawed in warm water at 40 to 45 °C. The first colostrum consumption took place between 1 and 3 h after birth and the second between 7 and 15 h after the first feeding. A sample of colostrum offered to every calf was frozen for subsequent determination of IgG concentrations. Thereafter, calves were bottle-fed 3 L of a MR containing (24% protein and 20% fat on a DM basis) diluted at 15% DM at each of the 2 daily feedings until the end of the study at 42 d of life. A starter pelleted concentrate (20% CP, 15.2% NDF, and 3.8% fat, on a DM basis) was fed ad libitum (50 g/calf at day 1 and progressively increasing by 20% of the consumption registered on the previous day) for the entire duration of the study. All calves had access to a bucket containing chopped (~2.5 cm in length) barley straw throughout the study.

All animals were inspected on a daily basis for health afflictions. Daily consumption of the starter concentrate and MR (and colostrum on the first day) were individually monitored. Calves were body weighed using an electronic scale at birth and on a weekly basis thereafter. A sample of each of colostrum fed to each calf was obtained and frozen at −20 °C for subsequent determination of IgG concentrations. Calves were blood sampled from the jugular vein using evacuated tubes without additives (Vacutainer, Becton Dickinson, Madrid, Spain) at 0 (before colostrum consumption), at 12 and at 24 h of life. Blood samples were placed in the refrigerator at 4 °C for ~15 min to induce coagulation and after centrifugation at 2000× *g* for 10 min at 4 °C, serum was obtained and frozen at −20 °C until subsequent analyses. Both colostrum and serum IgG concentrations were determined using radial immunodiffusion with a commercial kit (Bovine IgG Test Kit, Eurovet Veterinaria, Daganzo, Spain).

A power calculation using 3 means of 19, 21, and 22 mg of IgG/mL of plasma with a standard deviation of 1.6 and a power of 80% was ran and indicated that a minimum of 6 calves per treatment group would suffice to detect statistical differences at 0.05. One calf was the experimental unit as treatments were applied individually. The apparent efficiency of absorption (AEA) was calculated as the proportion of IgG in blood relative to the IgG consumed via colostrum estimating the plasma volume using the BW of the calf as proposed by [24]. Data were analyzed using a mixed-effects model, with treatment, time, and their interaction as fixed effects, and calf and block as random effects. Performance data were analyzed using time as a repeated measure applying an autoregressive variance–covariance structure of first order (as it yielded the lowest Bayesian information criterion) and body weight of the calf at birth as a covariate. The same model was used for serum IgG concentrations and AEA but the chosen variance–covariance structure for the repeated measures included the variance components. For non-repeated data (i.e., final BW, colostrum IgG), a mixed-effects model included treatment as the main effect and block as a random effect.

## 3. Results

**Figure 1:**
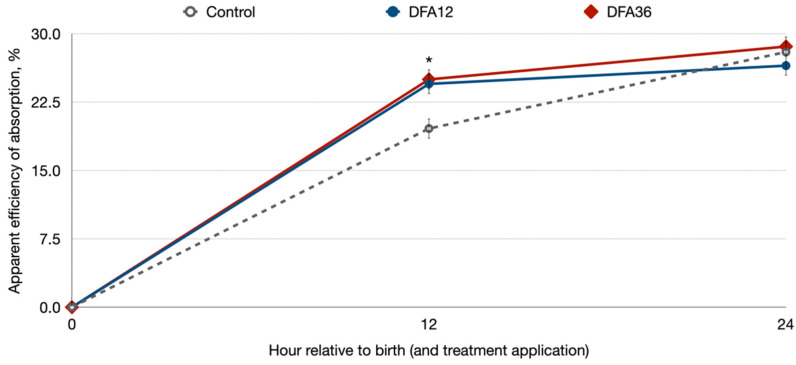
Serum IgG content (g) as influenced by treatment and time. The asterisk denotes differences between control vs. DFA12 and DFA36 at *p* < 0.05. Control: unsupplemented; DAF12: supplemented with 12 g/d of difructose anhydride III in the colostrum; DAF36: supplemented with 36 g/d of difructose anhydride III in the colostrum.

The overall health status of the animals in the current study was high, with 11 calves being treated for diarrhea (3 calves in control group, 3 in DFA12 group, and 5 calves in the DFA36 group). All cases of diarrhea were mild and all the animals recovered within 4 d. No differences in animal performance were found among the three treatment groups (Table 1). The mean colostrum IgG concentration fed in the current study was 110 ± 33.7 g/L (mean ± SD). Calves in all treatments consumed the same amount of colostrum with a similar concentration of IgG, and as a result, the amount of IgG consumed in both treatments was also similar (Table 2). Serum IgG concentrations were greater at 24 than at 12 h (Figure 1), but serum concentrations evolved similarly for the control, DFA12, and DFA36 calves with no differences among treatments. However, AEA was greater in DFA12 and DFA36 calves at 12 h of life than in control calves (Figure 1).

## 4. Discussion

The aim of this study was to assess whether DFAIII could provide additional advantages when supplemented to calves reared under ideal conditions (i.e., receiving high-quality colostrum and having a high health status). The growth performance and nutrient consumption in this study were relatively high. The lack of differences in performance among the treatment groups could be due to (1) a potential lack of effect of DFAII on performance, or (2) a relatively high degree of health and nutrient consumption that limited the possibilities to further improve performance. The IgG values in the colostrum reported in this study are in line with those found in previous studies [25] but greater than in others [26] and the commonly assumed concentration of 50 g of IgG/L. The high concentrations of the current study are due to the fact that only colostrum collected from dams withing the first 2 h after calving and those with a Brix value ≥ 32% were frozen and then used for feeding the calves enrolled in the experiment. It is well established that colostrum IgG concentrations progressively decline as time from calving increases [27].

The AEA observed herein (~25%) is in the low range reported in the literature, with most studies describing AEA around 30% [28,29]. Several studies have illustrated that the provision of high quantities of IgG may reach the full capacity of the intestine to absorb large molecules [30,31], and thus the relatively low AEA herein in comparison to previous studies could be partially due to the large amounts of IgG provided. Nevertheless, AEA was greater in DFA12 and DFA36 calves at 12 h of life than in the control calves (Figure 1), which suggests that DFAIII at a dose of 12 g/d may facilitate IgG absorption even when calves are fed high-quality (in terms of IgG content) colostrum and that greater supplementations (i.e., 36 g/d) provide no additional advantage. The mechanisms behind the improvement in EAE are unknown. Several authors have speculated that the increased absorption of IgG in calves supplemented with DFAIII could be due to an improvement in intestinal absorption through the paracellular pathway by increasing the opening of the epithelial tight junctions [22,32,33]. However, Jochims et al. [19] clearly illustrated that intestinal absorption of IgG in calves occurred through micropinocytic transfer. Thus, further research is needed to elucidate the potential reasons behind the improvement AEA of IgG when supplementing calves with DFAIII.

## 5. Conclusions

It is concluded that when feeding calves with high-quality colostrum, in terms of IgG concentration, supplementation with 12 g/d of difructose anhydride III may increase the apparent efficiency of absorption of immunoglobulins during the first 12 h of life of Holstein calves. However, no further consequences on growth performance or health status should be expected.

## Figures and Tables

**Table 1:** Performance of neonatal Holstein calves as affected by difructose anhydride III.

	Treatment ^1^				*p*-Value ^2^		
	Control	DFA12	DFA36	SE ^3^	T	t	Txt
Initial BW, kg	42.3	42.0	42.3	0.87	0.97	-	-
Final BW, kg (42 d of life)	71.4	71.8	70.2	1.51	0.76	-	-
Milk replacer intake, g/d	888	890	886	1.44	0.16	<0.01	0.35
Starter intake, g/d	274	303	247	32.1	0.46	<0.01	0.49
Dry matter intake, g/d	969	1004	941	38.3	0.51	<0.01	0.83
Average daily gain, g/d	707	717	690	27.6	0.78	<0.01	0.92
Feed efficiency, % ^4^	72.9	71.9	73.5	3.35	0.93	<0.01	0.81

^1^ Control: unsupplemented; DAF12: supplemented with 12 g/d of difructose anhydride III in the colostrum or in the milk replacer during the first 7 d of life; DAF36: supplemented with 36 g/d of difructose anhydride III in the colostrum or in the milk replacer during the first 7 d of life. ^2^ T: Effect of treatment; t: Effect of time, Txt: Effect of the interaction between treatment and time. ^3^ SE: Standard Error ^4^ Calculated as (average daily gain/total dry matter intake) × 100.

**Table 2:** Colostrum consumption, immunoglobulin content in colostrum and in serum, and apparent efficiency of absorption in neonatal Holstein calves as affected by difructose anhydride III supplementation.

	Treatment ^1^				*p*-Value ^2^		
	Control	DFA12	DFA36	SE ^3^	T	t	Txt
Colostrum consumed first 24 h, L	4.39	4.28	4.25	0.16	0.80	-	-
Colostrum IgG, g/L ^4^	116.0	116.3	96.23	0.05	0.58	-	-
IgG consumption first 24 h, g	508.9	498.0	409.2	1.12	0.77	-	-
Serum IgG, g/L ^4^	28.6	29.3	29.8	1.32	0.26	<0.01	0.66
AEA of IgG, % ^5^	23.4	25.4	26.7	1.05	0.20	<0.01	0.04

^1^ Control: unsupplemented; DAF12: supplemented with 12 g/d of difructose anhydride III in the colostrum or in the milk replacer during the first 7 d of life; DAF36: supplemented with 36 g/d of difructose anhydride III in the colostrum or in the milk replacer during the first 7 d of life. ^2^ T: Effect of treatment; t: Effect of time, Txt: Effect of the interaction between treatment and time. ^3^ SE: Standard Error. ^4^ Data were log-transformed. Means are back-transformed, whereas SE corresponds to log-transformed data. ^5^ AEA: Apparent efficiency of absorption. Calculated as Serum IgG (g/L) × 0.089 × BW (kg) ÷ IgG consumed (g) × 100.

## Data Availability

Data are contained within the article.
